# Dopamine Modulates Delta-Gamma Phase-Amplitude Coupling in the Prefrontal Cortex of Behaving Rats

**DOI:** 10.3389/fncir.2017.00029

**Published:** 2017-05-09

**Authors:** Victoria Andino-Pavlovsky, Annie C. Souza, Robson Scheffer-Teixeira, Adriano B. L. Tort, Roberto Etchenique, Sidarta Ribeiro

**Affiliations:** ^1^Departamento de Química Inorganica, Analítica y Química Física, Instituto de Química Física de los Materiales, Medio Ambiente y Energía (INQUIMAE), Facultad de Ciencias Exactas y Naturales, Universidad de Buenos AiresBuenos Aires, Argentina; ^2^Instituto do Cérebro, Federal University of Rio Grande do NorteNatal, Brazil

**Keywords:** LFP oscillation, comodulation, delta-gamma coupling, uncaging, dopamine

## Abstract

Dopamine release and phase-amplitude cross-frequency coupling (CFC) have independently been implicated in prefrontal cortex (PFC) functioning. To causally investigate whether dopamine release affects phase-amplitude comodulation between different frequencies in local field potentials (LFP) recorded from the medial PFC (mPFC) of behaving rats, we used RuBiDopa, a light-sensitive caged compound that releases the neurotransmitter dopamine when irradiated with visible light. LFP power did not change in any frequency band after the application of light-uncaged dopamine, but significantly strengthened phase-amplitude comodulation between delta and gamma oscillations. Saline did not exert significant changes, while injections of dopamine and RuBiDopa produced a slow increase in comodulation for several minutes after the injection. The results show that dopamine release in the medial PFC shifts phase-amplitude comodulation from theta-gamma to delta-gamma. Although being preliminary results due to the limitation of the low number of animals present in this study, our findings suggest that dopamine-mediated modification of the frequencies involved in comodulation could be a mechanism by which this neurotransmitter regulates functioning in mPFC.

## Introduction

Dopamine (DA) plays a central role in the executive functions undertaken by the prefrontal cortex (Björklund and Dunnett, 2007; Puig et al., 2014), with evidence of involvement in working memory (Chudasama and Robbins, 2004), decision-making (Schweimer and Hauber, 2006; Zhang et al., 2007), and inhibitory control (Navailles et al., 2014). However, physiological mechanisms underlying dopamine action in PFC remain elusive since its effect on different cell-types can be different and even opposite. Recent work proposed a two-stage mechanism by which dopamine D4 receptors (D4Rs) activation exerts opposite effects on pyramidal neurons and GABAergic interneurons in the PFC: (1) in the early phase DA release increases interneuron firing rate, leading to the suppression of PFC output signal; and (2) in the late phase DA promotes a global activity suppression over both pyramidal and interneuron, which could balance PFC network to basal levels (Zhong and Yan, 2016). This finding could explain the cortical hyper-excitability exhibited in D4R-deficient mice (Rubinstein et al., 2001) and provide a physiological mechanism of action for DA on PFC networks. Furthermore, it has been proposed that GABAergic inhibitory interneurons activity in the neocortex has a critical involvement in the generation of rhythms in different frequency bands (McBain and Fisahn, 2001). Yet, the effects of dopamine release on PFC neural activity remain hard to measure directly. The interpretation of electrophysiological recordings after intracerebral dopamine injection is complicated by time lags and concentration gradients related to diffusion, tissue damage, and other caveats. Optogenetic experiments offer a much better alternative, with millisecond precision and cell-mediated release (Tye and Deisseroth, 2012; Rosen et al., 2015). Another method of interest is the light-induced uncaging of dopamine, which we have recently developed as a novel technique for the fast delivery of dopamine in living brain slices (Araya et al., 2013). The technique employs RuBiDopa, a two-photon caged dopamine compound based on ruthenium-bipyridine chemistry that allows for very fast dopamine application by visible light irradiation. These caged compounds (RuBi compounds) release a neurotransmitter with an uncaging time in the nano-second scale and can be used with two-photon (2P) excitation (Filevich et al., 2010; Salierno et al., 2010), thus providing administration of molecules of interest with high temporal and spatial resolution. Until now, this new tool has only been used *in vitro*. Here we set out to assess the functionality of RuBiDopa on LFP recorded from freely-behaving rats. Other important improvement that optical methods present over the exogenous injection of neurotransmitter is that the latter, due to diffusion limitations, does not correctly represent physiological conditions, while the rapid activation of receptors via optical methods yields activation timing that mimics the tonic (slow) or phasic (fast) release of neurotransmitters (Rosen et al., 2015). Tonic and phasic DA release are underlied by physiologically distinct mechanisms. For instance, while dopaminergic neurons releasing tonic levels of DA (yielding concentrations in the nM scale) are under the influence of GABAergic inhibition, phasic DA release depends on modulation by glutamatergic inputs and yields a much higher, transient DA concentration (hundreds of μM–mM range; Goto et al., 2007). Although dopamine uncaging from RuBiDopa does not mimic synaptic conditions because of the need of diffusion from uncaging site to receptors and subsequent slower clearance of neurotransmitter (which also depends on diffusion), the kind of DA action obtained through this method is physiologically comparable to dopamine receptors activation with tonic dynamics.

LFP oscillations are typically classified into different frequency ranges and have been linked to a myriad of cognitive functions (Buzsáki and Draguhn, 2004; Buzsaki, 2006). The delta band (1–4 Hz) is a staple of slow-wave sleep (SWS) (Hobson and Pace-Schott, 2002), as its amplitude increases to the highest levels in this stage of the sleep-wake cycle. The theta band (5–12 Hz) has been extensively studied in the rodent hippocampus in relation to rapid-eye-movement (REM) sleep (Whishaw and Vanderwolf, 1973; Winson, 1974), spatial navigation (O'Keefe, 1976; Pavlides and Winson, 1989), and memory (Louie and Wilson, 2001; Hasselmo et al., 2002). Theta oscillations are also present in other cortical regions, including the PFC. Importantly, the amplitude of theta oscillations in the PFC and its coupling with hippocampal theta has been related with spatial memory in rodents (Jones and Wilson, 2005; O'Neill et al., 2013) and humans (Canolty et al., 2006; Ferreira et al., 2014; Kaplan et al., 2014). Gamma oscillations (>20 Hz) have been implicated in episodic memory in humans (Nyhus and Curran, 2010), as well as memory encoding and retrieval in the hippocampus of rodents (Colgin and Moser, 2010).

As new analytical tools are developed, it becomes increasingly clear that LFP oscillations can interact in multiple, non-excludent ways (Onslow et al., 2011; Lisman and Jensen, 2013). An interaction of particular interest is phase-amplitude comodulation, which occurs when the amplitude of a higher frequency oscillation is modulated by the phase of a slower rhythm (Tort et al., 2008, 2010; Canolty and Knight, 2010). This phenomenon has been studied in the hippocampus of behaving animals undergoing learning and memory retrieval (Tort et al., 2009), decision-making and working memory (Tort et al., 2008; Axmacher et al., 2010). In this brain region, theta-gamma coupling positively correlates with rodent's performance while learning a new task (Tort et al., 2009) and increases during working memory or retrieval demands, as in the central arm of the T-maze during alternation (Schomburg et al., 2014) and cued tasks (Tort et al., 2008). In human hippocampus, it has been proposed that comodulation plays a role in working memory by representing different items in gamma sub-cycles temporally ordered inside theta oscillations (Axmacher et al., 2010). And Kaplan et al. (2014) also found that theta-gamma coupling between theta phase from mPFC and gamma amplitude of the medial parietal cortex increases during retrieval of a spatial memory. These results suggest that cortical networks in the mammalian brain actively use comodulations to perform neural computation. The role of DA in neural phase signaling has been studied, showing that this neuromodulator plays a key role in triggering and maintenance of high-coherence states of LFP bands across brain regions. Infusion of DA in PFC increased coherence in the theta range between PFC and hippocampus CA1 region (Benchenane et al., 2010). Moreover, coherence between these two areas is impaired in a genetic model of schizophrenia, a pathology highly related to DA dysfunction (Sigurdsson et al., 2010). Mice with hyperdopaminergia showed increased hippocampus-PFC gamma phase synchrony (Dzirasa et al., 2009), while a DA receptor antagonist attenuated theta phase coupling and theta-gamma phase-amplitude comodulation between these regions (Xu et al., 2016).

Given the well-known implication of DA in PFC functioning in health conditions and disease and increasing evidence over the last years about frequency coupling playing a role in different key processes of neural functions, we aimed to study the presence and mechanisms underlying coupling in this brain region. In this work we specifically aimed to probe theta-gamma comodulation in the PFC and whether it is modulated by dopamine. To this end, we performed electrophysiological recordings in the medial PFC of freely behaving animals chronically implanted with multielectrode arrays. Dopamine was uncaged using green light delivered by a fiber optic to the mPFC, and its effect was measured as changes in the theta-gamma and delta-gamma comodulations.

## Materials and methods

### Chemical synthesis

All reagents were obtained from Sigma-Aldrich and used without further purification. The precursor complex [Ru(bpy)_2_(PMe_3_)Cl)]PF_6_ was synthesized as previously described (Salierno et al., 2010): 520 mg of [Ru(bpy)_2_Cl_2_] (Sigma-Aldrich) was added to 20 mL of methanol/water solution (1:1) and refluxed under N_2_. Then a solution of trimethylphosphine in THF (Tetrahydrofuran, 1.2 mL of 1 M solution) was added by syringe. The reaction was followed by observation of UV–Visible (UV–Vis) spectra. In some cases, additional phosphine solution was added. Once the UV–Vis spectrum was stable, methanol and excess phosphine were removed by vacuum distillation. The resulting aqueous solution was filtered to remove any solids, and precipitated by the addition of excess of KPF_6_ at 0°C. The resulting dark orange solid was washed three times with cold water and dried. This precursor was solubilized in water by adding 100 mg of [Ru(bpy)_2_(PMe_3_)Cl)]PF_6_ to 3 mL acetone, and 3 mL of an aqueous suspension of 1 g of anionic resin Dowex-22 (chloride form). Before removing acetone by rotary evaporation, precursor was left in Dowex 22 for 10 min to allow PF_6_/Cl exchange. Then, 100 mg of dopamine.HCl were dissolved in the precursor complex solution under nitrogen atmosphere. After bubbling of N_2_ for 15 min, 2.5 equivalents (50 mg) of solid NaOH were added. The solution was heated under N_2_ during 16 h to prevent dopamine oxidation, cooled to 0°C and neutralized with 120 μL of acetic acid. In order to remove insoluble material, the solution was filtered and poured over excess of saturated solution of KPF_6_ to precipitate RuBiDopa. The product was purified by redissolving three times in an aqueous suspension of Dowex-Cl and further reprecipitation with KPF_6_. The redissolution/precipitation cycle was performed three times, and the aqueous solution obtained was lyophilized in dark, yielding a very hygroscopic orange powder. Synthesis process was performed in the dark to avoid photolysis of the complex.

It should be noted that it is not possible to perform a negative control injecting the caging component only, without DA, for this “caging component” does not exist as is. These Ruthenium complexes have 6 coordination positions and all 6 positions are occupied by a ligand in any stable complex. Therefore, one can replace other ligand for dopamine, but not leave it “empty.” As any replacement will change some chemical properties (charge, hydro/lipophilicity, electron density, etc.) would not be a good choice for a control.

### Animals

Three male Wistar rats (2–3 months age, 250–350 g) obtained from our breeding colony were used for multiple neurophysiological recording sessions. Animals were kept under a 12-h light-dark cycle (lights on at 07:00) with food and water *ad libitum*. All animal care including housing, surgical, and recording procedures were approved by the Ethics Committee for Animal Use of the Federal University of Rio Grande do Norte (permit # 11/2015 CEUA-UFRN).

### Surgical implantation of multielectrode arrays

**Figure 1:**
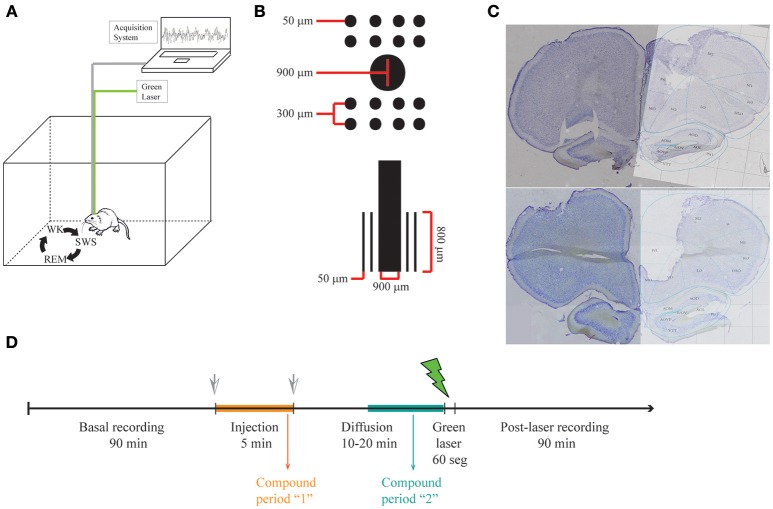
**LFP recordings in freely behaving rats. (A)** Illustrative representation of recording setup. While recording LFP from mPFC in freely behaving rats, sleep-wake cycle was monitored by video recording in a familiar arena. **(B)** Scheme of the multielectrode arrays with a central cannula. **(C)** Histology showing electrode lesions in the target area (medial Prefrontal Cortex Prelimbic area, PrL). **(D)** Experimental protocol.

Animals were anesthetized with ketamine and xylazine and implanted with multiple 50 μm Teflon-coated tungsten microwires (electrode spacing: 300 μm; impedance: ~1 MΩ at 1 kHz) partitioned in two 2 × 4 arrays with a 900 μm diameter cannula in the middle (Figure 1B).

### Electrophysiological recordings

Animals were allowed for recovery for 1 week before recordings were performed. Experiments were carried out in the afternoon and animals were allowed to spontaneously traverse the sleep-wake cycle (Figure 1A). Animals were freely behaving in all our experiments, since this procedure gives the experimenters the big advantage of recording neuronal activity in a more naturalistic way, without the influence of anesthetics in the underlying physiology. During each recording session, animals were placed in a rectangular chamber (60 × 40 × 40 cm) at a dimly lighted room. Recording sessions (up to 3 h) included continuous video and electrophysiological recordings using a RHA2000 data acquisition board, a RHA2116 pre-amplifier and PC-interface software (Intan Technologies, USA). LFPs were preamplified (1,000x), filtered (0.7–300 Hz), and digitized at 1,000 Hz. Reference electrode (silver wire, bared, 200 μm diameter) was soldered to a screw and positioned on the skull's right-posterior bone. The array was placed in the mPFC (centered at anteroposterior (AP): 2.95 mm and mediolateral (ML): 0.5 mm from Bregma; and dorsoventral (DV): –4 mm from the pial surface). The final position of electrodes is Prelimbic mPFC (layers II, III). The placement of electrodes in the mPFC was confirmed by inspecting coronal brain sections stained with cresyl violet (Figure 1C).

### Rubidopa administration and uncaging

The multielectrode arrays were mounted with a cannula in the center, through which solutions were injected and a fiber optic was placed to uncage dopamine from RuBiDopa (Figure 1B). After ~1.5 h of baseline recording, 5 μl of a 300 μM RuBiDopa solution was applied using an injection system (cannula, silicone tubing, Hamilton syringe, and pump) at a rate of 1 μl/min. The injection cannula was left in place for additional 10–20 min to allow further diffusion of RuBiDopa, then it was replaced by the fiber optic to deliver green light (~1 mW) (Figure 1D). It was not possible to keep this time window identical for all animals due to the difficulty of properly placing the fiber optic on freely moving animals. Also, this procedure implies that the animal wakes up in case it was in a sleep stage (SWS or REM sleep), so the recording corresponding to the uncaging period will be in the wake stage of the sleep-wake cycle. A doubled Nd-YAG DPDSS laser (532 nm, 5 mW) was used to uncage dopamine from RuBiDopa. The light power on sample was 1 mW. The module was coupled through a 5 mm lens to a multimode fiber optic (250 μm outer diameter), which was introduced into the cannula after the injection was performed. The laser power supply was controlled by a low-current remote switch. To keep 250 μm fiber optic in place inside 900 μm cannula and prevent the fiber from changing its position, another cannula was fixed at the end of the fiber (25 gauge, 260 μm ID, and 510 μm OD), which was placed in the array center cannula (20 gauge, 600 μm ID, and 900 μm OD). To avoid injection cannula or fiber optic from getting out of the center cannula due to potential movements of the animal, injection tubing and fiber optic were fixed trough a piece of masking tape to the head-fixed Intan headstage during injection and uncaging, respectively. The light power used for uncaging (1 mW) saturates the uncaging capacity of dopamine from RuBiDopa at the concentration indicated in the experiment. This way possible differences in light intensity reaching the electrodes are not expected to interfere with dopamine concentrations reached around them.

### Data analysis

All analyses were performed using customized code in MATLAB (MathWorks, Natick, MA). For group analysis, all electrodes of the matrix were used (excluding electrodes that presented artifacts, which differed between animals), while for figures where data from one representative electrode is shown, we selected the channels with the highest values of comodulation.

### Estimation of phase-amplitude comodulation

**Figure 2:**
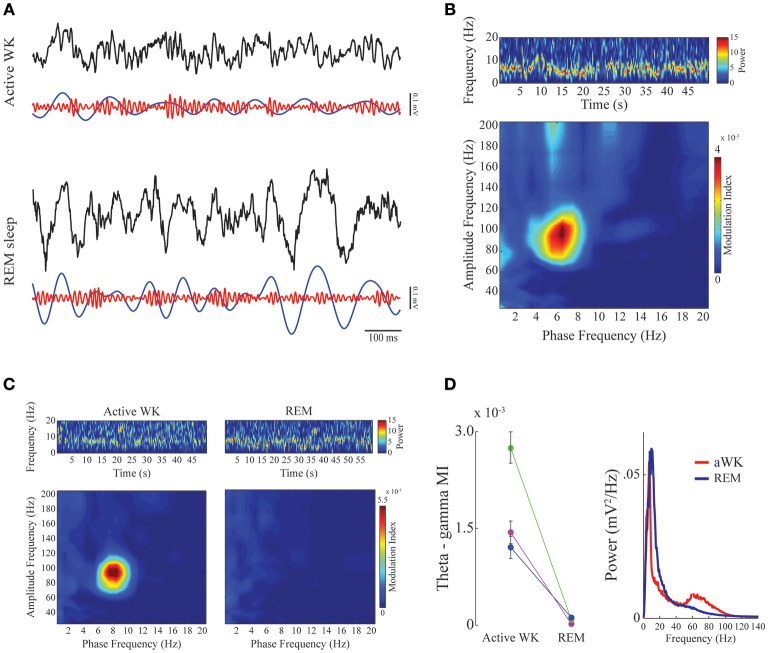
**Phase-amplitude CFC between theta and gamma in mPFC during active waking and REM sleep. (A)** Panels show 1-s of raw (black), theta-filtered (blue, 5–12 Hz), and gamma-filtered (red, 60–110 Hz) LFPs for active waking (top) and REM sleep (bottom) stages. Notice consistent CFC during active WK, in which gamma amplitude waxes and wanes depending on the phase of the theta cycle, and exhibits highest amplitude at theta descending phase. **(B)** Spectrogram (top) shows prominent theta activity during a representative period of active waking. Phase-amplitude comodulogram (bottom) computed for the same period, revealing high CFC between theta phase and the amplitude of gamma. Spectrogram color scale is in mV^2^/Hz. **(C)** Spectrograms showing prominent theta activity during both active waking (left) and REM sleep (right). The bottom panels show comodulograms computed for the respective periods. Spectrogram color scale is in mV^2^/Hz. **(D)** Left panel: Group result (*N* = 3) taking all high theta periods and sorting them according to sleep-wake stages. Different colors represent different animals. Modulation index for theta-gamma during active WK period was significantly different from REM sleep in all cases (Friedman's test, *p* < 0,05). Error bars represent standard error of the mean (SEM, *N* = number of channels for each animal; purple, magenta: 14 channels; green: 13 channels). Right panel: mean power spectral density for the epochs used in the left panel. Notice a small bump in the gamma range only for PSD obtained during active waking.

**Figure 3:**
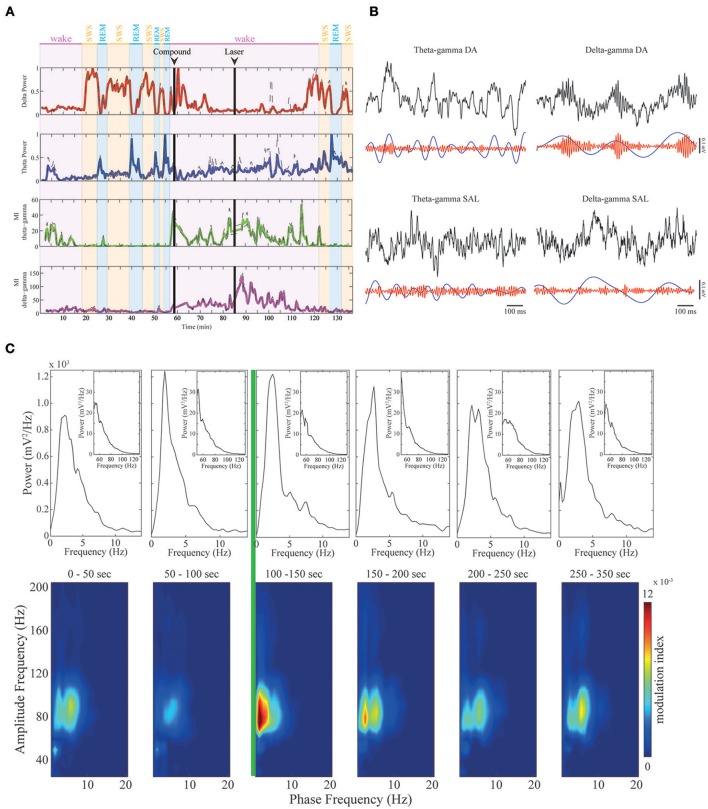
**Dopamine modulates CFC in mPFC. (A)** Upper panels: delta (red trace) and theta (blue trace) band power across sleep-wake cycles in a representative animal. Delta power is high in SWS, while theta power is high during REM sleep and active WK periods. Lower panels: continuous modulation index during the whole experiment. As shown before, theta-gamma comodulation (green trace) only appears in active wake and is absent during REM sleep, while delta-gamma comodulation (magenta trace) is absent before RuBiDopa injection. After dopamine uncaging, the strength of the delta-gamma comodulation significantly increases, and then it returns to basal values. **(B)** Examples of raw data for theta-gamma and delta-gamma coupling in the presence of DA (after RuBiDopa uncaging) and in control conditions (after saline injection). In all cases, upper black traces represent 1-s period of recording of raw LFP. Lower traces represent filtered frequency bands. As noticed, in DA condition the amplitude of gamma (red trace) is modulated by the phase of delta (blue trace), with gamma having high amplitude during delta peaks/descending phase. In the others conditions we do not find correlation between gamma amplitude and theta/delta phase. **(C)** Peri-event Power Spectrum Density plots (PSDs) and comodulograms. Upper panels: power spectra of 50 s-epochs for delta/theta bands (inset: gamma band). Below, six comodulograms for the respective 50 s epochs. The green line represents laser onset, leading to RuBiDopa uncaging.

Phase-amplitude comodulation between two oscillations of different frequencies was assessed as described in Tort et al. (2008, 2010) and Scheffer-Teixeira et al. (2012). Briefly, this analysis measures the comodulation between the amplitude of the faster frequency and the phase of the slower frequency. Both amplitude and phase series are obtained first by filtering the LFP in the desired band frequency (e.g., theta: 4–12 Hz and gamma: 60–100 Hz) and then applying the hilbert function to obtain its analytic representation; from this transformed signal, instantaneous phase and amplitude series are extracted. A Modulation Index (MI) is computed from the mean amplitude distribution over phase bins (18 phase bins of equal size were used in this work): while a uniform distribution indicates lack of comodulation between phase and amplitude, deviations from the uniform distributions indicate the presence of phase–amplitude comodulation (Tort et al., 2010). To quantify these deviations, the MI is based on information theory and uses a normalized version of the Kullback-Leibler distance, which calculates the information gain (in terms of Shannon entropy) when using one distribution (observed phase—amplitude) instead of a more parsimonious one (uniform distribution). Based on Tort et al. (2008), we first apply the Shannon entropy

$$\begin{array}{l} H=-\sum_{j=1}^{N}p_{j}\;log\;p_{j}\; \end{array}$$

where N is the total number of phase bins (18 bins used in this work). Also, p_*j*_ is the normalized mean amplitude in bin *j* given by

$$\begin{array}{l} p_{j}=\;\frac{<A_{f}>\left(j\right)}{\sum_{j=1}^{N}<A_{f}>\left(j\right)} \end{array}$$

where A_*f*_ is the amplitude series of the filtered signal (*f* denotes the center frequency). And finally, the MI is calculated as

$$\begin{array}{l} MI=\;\frac{H_{max}-H}{H_{max}} \end{array}$$

where H_max_ is obtained from the uniform distribution, in which p_*j*_ = 1/N and then, H_max_ = log N. Therefore, MI-values range between 0 and 1; due to the normalization, low entropy values (H) result in high MI-values. The MI is only computed for one frequency pair; a comodulation map, or comodulogram (as in Figures 2, 3, Supplementary Figures 1–3) is constructed by scanning for coupling across several frequency pairs and expressing MI-values by means of a 2D heatmap. We also used the terms theta—gamma or delta—gamma to denote the MI calculated only for this frequency pair. In order to control for possible artifactual origins of phase-amplitude islands in the comodulogram, three criteria were used: (1) visual inspection of the raw signal should clearly show the presence of phase—amplitude coupling (a faster oscillation should increase its amplitude in a specific part of the cycle of a slower and dominating wave; this is shown in Figures 2A, 3B) and (2) the absence of sharp deflections; also (3) the original comodulogram values should be higher than 97.5th-ile of a distribution of surrogates comodulograms. For this latter analysis, the comodulogram was exactly calculated as described before, but with one difference: before obtaining the amplitude series, the signal was randomly split, both parts were exchanged and concatenated again. Finally, an observed *p*-value is calculated by comparing the original x-y frequency pair entry in the comodulogram against a distribution of 300 surrogates. Non-significant values were set to zero (dark blue color) in the final comodulogram (Supplementary Figure 2).

**Figure 4:**
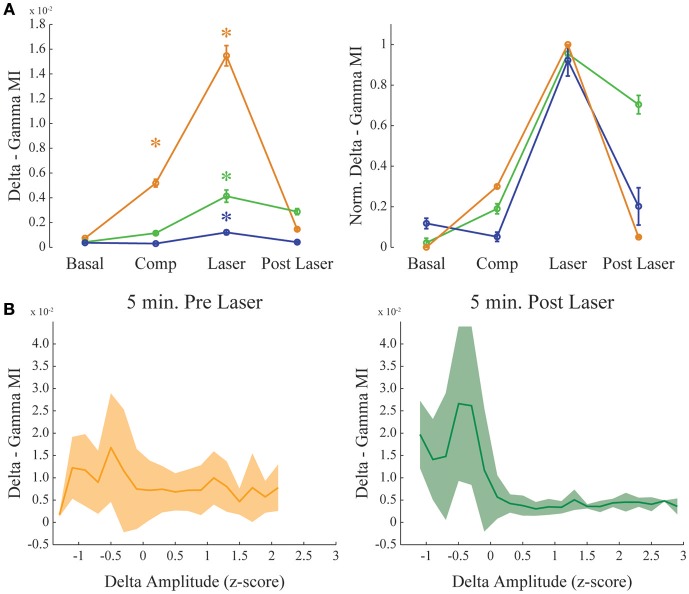
**Dopamine increases delta-gamma comodulation. (A)** Group result showing raw (left) and normalized modulation index (right) for the delta-gamma comodulation in the 4 different conditions of the experiment. Basal: recording immediately before beginning the injection. Comp: 10 min after compound injection. Laser: 1 min irradiation. Post Laser: 10 min after laser irradiation. Each color represents one animal, and all recording channels were pooled together per recording session. Friedman's statistical test, Tukey's *post-hoc* test (*p* < 0.05). Error bars represent SEM (*N* = number of channels for each animal; orange: 14 channels; green: 13 channels; blue: 10 channels). Asterisks represent statistically significant differences in comparison with basal recordings. **(B)** Group results comparing delta—gamma coupling 5 min before and after laser onset while controlling for normalized delta amplitude. Solid line represents the mean and shaded area the standard deviation.

**Figure 5:**
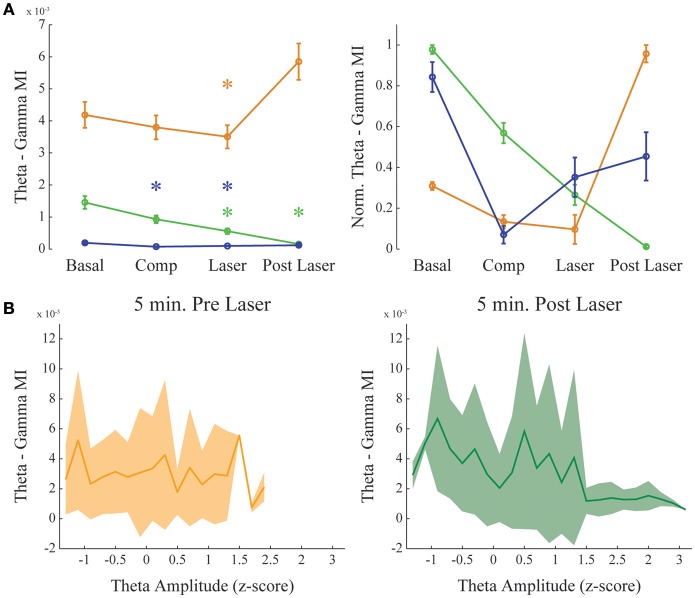
**Dopamine uncaging has no effect in theta-gamma comodulation level**. Same as in Figure 4, but for theta—gamma coupling. **(A)** Group result showing raw (left) and normalized modulation index (right) for the theta-gamma comodulation in the 4 experimental conditions. “Basal” and “Post-Laser” conditions were calculated from periods of active waking in the corresponding part of the experiment, to allow analysis of theta-gamma comodulation. Comp: 10 min after compound injection. Laser: 1 min irradiation. Each color represents one animal, and all recording channels were pooled together per recording session. Friedman's statistical test, Tukey's *post-hoc* test. (*p* < 0.05). Error bars represent SEM (*N* = number of channels for each animal; orange: 14 channels; green: 13 channels; blue: 10 channels). Asterisks represent statistically significant differences in comparison with basal recordings. **(B)** Group results comparing theta—gamma coupling 5 min before and after laser onset while controlling for normalized theta amplitude. Solid line represents the mean and shaded area the standard deviation.

The time course of comodulation strength shown in the third and fourth panels of Figure 3A was obtained by building gray-colored comodulograms and then processing them stacked with the Z-project function of ImageJ software, using 50-s windows with 25-s overlap. For group results reported in Figures 4, 5, comodulation strength was calculated for 5-min periods corresponding to different stages in the experiment. Basal recordings were defined as the period before the beginning of the injection. In the panels of Figures 4A, 5A, the Compound period corresponds to the interval 10–15 min after the beginning of the injection. For Supplementary Figure 5, quiet waking and active waking periods were selected inside the 5 min window around laser onset. In Figures 4, 5, the error bars correspond to different electrodes in the same animal.

### Filter settings, amplitude, and phase time series extraction, spectral analyses

Filtering was performed using *eegfilt* function from the EEGLAB toolbox (Delorme and Makeig, 2004). After filtering, amplitude and phase time series were obtained from the analytical representation of the filtered signal by means of the Hilbert transform (*hilbert* function from the Signal Processing Toolbox). Time-frequency spectral decompositions were obtained by using the *spectrogram* function from the Signal Processing Toolbox, and power spectral densities (PSD) were calculated using the *pwelch* function from the same toolbox. Figure 3A shows the mean instantaneous amplitude (mean over channels) of delta- and theta-filtered signals, normalized to achieve values between 0 and 1 (i.e., NormAmp = (Amp-min(Amp))/(max(Amp)-min(Amp))). This same normalization procedure was also used to obtain the MI in Figures 4A, 5A (right panels). In Figures 4B, 5B, Supplementary Figure 3 (right panel) we obtained the power of the referred frequencies (delta or theta) and the modulation index (delta or theta and gamma) for consecutive 5 s time windows (with 2.5 s overlap). For each animal, the power obtained in each time window was normalized (z-scored) and pooled among them. The delta, theta, and gamma bands were respectively defined as 0–4, 5–12, and 60–110 Hz.

### Estimation of REM sleep, slow wave sleep, active waking periods, and quiet waking

All periods were defined by spectral analysis combined with visual inspection of the video recordings. REM epochs were defined as periods of quiescence, REM sleep posture, and elevated theta power (theta/delta > 4), while SWS epochs were defined as periods of quiescence, SWS posture and low theta power (theta/delta < 4). Active waking periods were obtained by visual inspection of locomotion behavior and dominating theta oscillations, while quiet waking epochs were obtained as absence of exploratory behavior, clear signs of waking, and absence of theta oscillations.

## Results

We characterized phase—amplitude coupling patterns of the LFP recorded in the mPFC during spontaneous behaviors and under the influence of dopamine uncaging.

### Theta-phase modulates the gamma band in the mPFC in a state-specific manner

We found a high level of comodulation between theta phase and a gamma sub-band (60–110 Hz) in the mPFC. Figure 2 (A upper panel and B) shows the presence of phase-amplitude CFC only during active waking period, but not during REM sleep. Both states are associated to the constant presence of a theta oscillation dominating the spectrum (Figures 2B,C, upper spectrogram panels; also see Supplementary Figure 1 for individual examples). Previous work in the hippocampus has shown that theta phase modulation of gamma amplitude is highly dependent on theta power; comodulation is present whenever theta power is high, as during active waking and REM sleep periods (Scheffer-Teixeira et al., 2012). The modulation index in the mPFC recordings during theta-rich periods shows that theta-gamma comodulation was present during waking, but completely disappeared during REM sleep, despite the fact that theta amplitude is high in both stages (Figures 2C,D, right panel). Group data confirms that theta-gamma comodulation was stronger during waking than during REM sleep (Figure 2D, “Active WK” is significantly different from “REM” in all cases. Each color represents one animal; *p* < 0.05, Friedman's test). We also found evidence that gamma oscillations are reduced or even absent during REM sleep (Figure 2D, right panel), which explains the lack of theta-gamma coupling during this state. Figure 3A shows LFP power variations across time during an experimental session (upper panels, red and blue traces for the delta and theta bands, respectively). By our definition of sleep-waking states (see Methods), when the animal assumes sleep posture we expect maximum delta band power during SWS and theta band power during REM sleep; during waking periods, however, theta oscillations are also present, but mainly during active spatial exploration. Notice that theta-gamma coupling dynamics can also be inferred by this graphic: green trace represents the magnitude of theta-gamma comodulation, which only appeared during active waking (Figure 3A).

### Delta-gamma comodulation in mPFC is modified by dopamine

Dopamine uncaging by light did not produce overt behavioral changes. Interestingly, after dopamine uncaging, the strength of the delta-gamma comodulation significantly increased when compared to the other conditions of the experiment (representative example shown in Figure 3A, magenta trace). Figure 3B shows examples of raw data for DA and control conditions (saline) for theta-gamma and delta-gamma frequencies. As noticed, in the presence of DA delta and gamma bands, represented by blue and red traces respectively, are correlated: gamma transient bursts occur in the peak/descending part of delta oscillations. This coupling phenomenon is absent during quiet waking (Supplementary Figure 1) and despite present during SWS, it shows a reduced coupling strength (Supplementary Figure 3). Comodulograms before and after the activation of RuBiDopa (Figure 3C, lower panels) show the appearance of strong delta-gamma comodulation immediately after light activation (green line). This comodulation was absent in the baseline recordings and remained at low levels after compound injection in the absence of green light. The power spectral densities (Figure 3C, upper panels) show that delta or gamma power changes does not explain the delta-gamma coupling observed after laser onset (e.g., second panel from left to right shows the same delta and gamma power as the panel after the green laser). Supplementary Figure 4A shows group PSDs for all the channels involved in the analysis. Black and red traces representing pre and post-laser epochs, respectively, shows overtly the same delta and gamma power for both periods. When extracting the power bands of interest per animal, we found some significant values (Supplementary Figure 4B). Nevertheless, the size of this effect is small, and importantly, the subtle effect found in power is a decrease, suggesting that delta-gamma coupling may appear because of system changes caused by dopamine uncaging, and not just as a power gain.

Figure 4 shows delta-gamma results for all animals following RuBiDopa activation. We found a significant increase in delta-gamma modulation index after compound activation when compared to basal recording (Figure 4A). Moreover, delta-gamma MI was also higher after the laser onset while controlling for delta amplitude (Figure 3A red and magenta traces and Figure 4B) and when using only quiet waking epochs, a delta-associated behavioral state (Supplementary Figure 5, left panel). When RuBiDopa was injected in the mPFC before activating it with light, we found an effect of RuBiDopa alone (Figure 4A). This effect can be attributed to caged dopamine having certain ability to activate dopamine receptors, or to the presence of free dopamine traces in the RuBiDopa solution. As can be noted from Figure 4A, the three animals presented in this work showed different basal values of delta-gamma coupling and moreover, different levels of change (increase) in these values after dopamine uncaging. These differences can be attributed to natural variability between animals. Nevertheless, in all cases there is a significant increase in delta-gamma coupling strength after light stimulation and the effect is comparable when values are normalized (Figure 4A, right).

As it was shown before (Figure 2), theta-gamma coupling is present in mPFC in a state-specific manner, appearing only during active waking periods. We further investigated if theta-gamma coupling during WK state was also modified by the presence of DA after RuBiDopa uncaging. Figure 5A shows group results on theta-gamma comodulation for all the experimental conditions of the RuBiDopa experiment. MI levels were quite variable from one experiment to another; overall, we concluded that theta-gamma comodulation is not significantly affected by RuBiDopa administration and further activation by light (see also Figure 3A blue and green traces showing little changes). We also see little changes in theta—gamma coupling before and after laser onset while controlling for theta amplitude (Figure 5B) or even when using only epochs from active waking, a theta-associated behavioral state (Supplementary Figure 5, right panel). In these analyses, we used 5 min around laser activation.

## Discussion

Our results suggest that theta phase modulates the amplitude of gamma oscillations in the mPFC. Also, this modulation is state-dependent, only occurring during active waking; during REM sleep we observed the absence of theta-gamma coupling and reduced power in gamma band (Figure 2D). And finally, it does not depend on the theta amplitude (Figure 5B). We also found that dopamine uncaging by light activation produces a sudden change in the coupling of gamma oscillations, which become modulated by the phase of delta oscillations rather than by the theta rhythm phase. We did not find strong delta-gamma coupling during other states also associated to high delta power, as SWS (Supplementary Figure 3) and quiet waking (Supplementary Figure 1). It should be noticed that the comodulation seen during SWS (Supplementary Figure 3), despite present, is not structured into a clear comodulation island and has a small effect size. We also showed that the observed increase in delta-gamma MI is not the result of an increase in the power of the frequency bands involved. For instance, PSDs of all the channels analyzed show no changes before and after laser onset, and subtle changes found when extracting the bands of interest for the different animals shows decreasing rather than increasing power. Also, it has to be noted that delta-gamma coupling is stronger for small delta amplitude values (Figure 4B), suggesting that the increase in MI does not involve a signal gain in the frequency band being modulated.

While the small sample size is an important limitation of the study, the data altogether suggests that the shift in comodulation from theta to delta observed after RuBiDopa uncaging can be attributed to the neurotransmitter dopamine acting on dopamine receptors in the mPFC. Another caveat worth mentioning is that theta-gamma comodulation was only present during the limited periods of time when the animals were actively exploring the environment. It is therefore difficult to reach a definite conclusion regarding dopamine-related changes in theta-gamma comodulation. On the other hand, the increase in delta-gamma comodulation caused by dopamine uncaging was quite robust. As it was stated in the Methods section, the experimental conditions reached in this work imply that the dopamine uncaging only can be done in the waking stage of the sleep-wake cycle. Therefore, it remains an open question if DA uncaging in the mPFC would also underlie delta-gamma coupling during sleep states.

Regarding the dopamine concentrations reached in these experiments, we did not expect differences in the effect observed in electrodes at different distances from the fiber, since we applied light of such power so as to saturate the uncaging capacity of dopamine from RuBiDopa at the concentration indicated in the experiment. Hence, possible differences in light intensity reaching the electrodes did not interfere with dopamine concentrations reached around them. Regarding kinetics and concentration of DA in physiological conditions, this is an issue that have been controversial over the years, as there has not been consensus about the factors that govern and determine DA concentrations in DA synapses. As most DARs are located extrasinaptically, DA needs to “spillover” the synaptic release site to be able to act on receptors. This spillover is known to be mediated by different mechanisms such as uptake by DA transporters (DATs) and diffusion. The kinetics of these two processes compete and diffusion seems to have more influence in determining the net extracellular concentration of DA and thus the amount of DA that reaches DARs (Cragg and Rice, 2004). Factors such as diffusion rate, distance between release site and receptors, and also DATs-mediated uptake, will determine if the amount of DA present at the DAR is sufficient to activate it and gate a response. *In vitro* studies determining affinity constants and EC_50_ values for DARs have shown that receptor activation occurs in a range between 10 nM (high-affinity states) and 1 μM (low-affinity states; Richfield et al., 1989; Neve and Neve, 1997). The final dopamine concentration in the present experiments is difficult to calculate properly because of lack of control of diffusion through the brain tissue. Nevertheless, taking into account the diffusion factor from injection site to electrodes (which we roughly estimate in a factor of 10), and knowing that with a light intensity of 1 mW (see Methods) all molecules of RuBiDopa will release DA, we estimate the DA concentration reached in this work to be in a value between 25 and 30 μM. Thus, this concentration should be highly efficient in activating DARs in low as in high-affinity states. It is well-established that DA release from dopaminergic neurons can be slow, low-amplitude and constant (tonic) or fast, high-amplitude and transient (phasic). These two mechanisms are physiologically different and importantly, can trigger opposite responses in postsynaptic neurons (Rosen et al., 2015). The kind of DA action obtained through RuBiDopa uncaging in this work is physiologically comparable to dopamine receptors activation with tonic dynamics. Physiologically, there are significant differences in DA concentration intrasynaptically (where 25–30 μM could be in the range or even lower) and extrasynaptically, where this represents a rather substantial increase beyond the normal 10–20 nM of this compartment. Therefore, this could be a constraint in the interpretation of the data, since concentrations reached in this work, despite being sufficient to activate DARs in extrasynaptic space, are also significantly higher than in physiological conditions.

Previous studies with RuBi compounds did not show any toxic effect at the concentration used in this work (300 μM) (Rial Verde et al., 2008; Lopes-dos-Santos et al., 2011; Araya et al., 2013). It should also be noted that the animals used in the present study showed normal LFP and spike recordings for up to a month (data not shown), which reflects intact brain functionality in the region where RuBiDopa was administered for experiments. This suggests that the caged compound did not affect physiology through a toxic effect. As stated before (see Methods), it is not possible to use an “empty” caging molecule as a negative control for our experiments. Nevertheless, future experiments administering DA antagonists remain necessary as a negative control.

Our work also suggests that frequency band power in the delta and gamma ranges does not significantly change as a result of DA uncaging. For instance, during REM sleep, theta power is high but theta-gamma coupling is absent (Figures 2C,D), and during SWS the same is valid for delta band (high delta power and low delta-gamma coupling; Figure 3A, Supplementary Figure 3). Figures 4B, 5B also show that delta and theta amplitude are not positively correlated with the comodulation strength in the mPFC respectively. Previous articles point to the importance of coupling strength alone in cognitive functions such as learning and memory retrieval, decision-making and working memory (Tort et al., 2008, 2009, 2010; Axmacher et al., 2010; Kaplan et al., 2014). Our results suggest that dopamine release in the mPFC could act not by increasing amplitude gain of slower oscillations, but by fine-tuning inhibitory networks (Zhong and Yan, 2016), which interact with principal excitatory cells (Tseng and O'Donnell, 2004) and generate gamma oscillations (McBain and Fisahn, 2001; Bartos et al., 2007).

It is known that abnormal dopamine activity plays a major role in the pathophysiology of schizophrenia (hyperdopaminergia in the mesostriatal pathway, frontal hypodopaminergia in the mesocortical pathway), and impaired neuronal synchronization within cortical networks in the gamma frequency band has been proposed to contribute to several psychiatric disorders also characterized by cognitive deficits. In the PFC, it has been proposed that dopamine modulates working memory capacity (Seamans and Yang, 2004). Dopamine has also been shown to modulate the gamma band, which is involved in cognitive function (Furth et al., 2013). Moreover, a shift in the phase-frequency of the comodulation to slower rhythms is involved in the increase in working memory capacity in the hippocampus (Axmacher et al., 2010). Shifting the modulating frequency of comodulation from theta to delta rhythms as a result from dopamine action can reflect synchronization features between mPFC and ventral tegmental area VTA. Peters et al. (2004) showed that VTA slow oscillations drive PFC pyramidal neurons up-states, and both structures are coherent in the delta range. Also, VTA inactivation by lidocaine injection lead to suppression of slow oscillations in the PFC. Therefore, dopamine uncaging in our work could mimic the dopaminergic component of the VTA-PFC projection. Our results are in accordance with previous results showing that DA has a key role in triggering and maintaining synchrony and coupling between different LFP bands and across different brain regions (Dzirasa et al., 2009; Benchenane et al., 2010; Sigurdsson et al., 2010; Xu et al., 2016).

The present data also constitute the first evidence of theta-gamma comodulation in the mPFC of freely behaving rats during active exploration, in agreement with recent results in mice (Zhang et al., 2016). Also, the lack of theta-gamma comodulation in the mPFC during REM sleep, despite the presence of theta rhythm generated in the hippocampus during this state, has recently been described (Zhang et al., 2016) and remains to be elucidated. Systemic features of REM sleep, such as the interruption of sensory inputs or the shut-down of the noradrenergic/serotonergic drive, are unlikely to explain this absence, because theta-gamma comodulation occurs during REM sleep in the hippocampus (Scheffzük et al., 2011; Scheffer-Teixeira et al., 2012). While hippocampal ripples and mPFC spindles episodes are synchronized during SWS (Siapas and Wilson, 1998), the present results along with the observations that spike-timing correlations between HPC and PFC decrease (Wierzynski et al., 2009) and theta—gamma coupling increases in the hippocampus during REM sleep (Scheffzük et al., 2011) suggest a decoupling event between both structures.

Plastic changes related to DA input may also underlie delta-gamma comodulation observed after laser activation of RubiDopa or DA injection. DA fibers terminals arising from VTA innerve the same area as hippocampal inputs to PFC (Carr and Sesack, 1996). Also, it was shown that DA injection and VTA stimulation modulate long term potentiation and depression in the hippocampal—PFA synapses (Jay et al., 1996, 2004; Gurden et al., 1999). Our current investigation does not allow us to completely elucidate which events are at play; further research, therefore, must be performed to address the exciting possibility that theta-to-delta coupling change may reflect a PFC gating mechanism for controlling external influences.

The low number of animals presented in this study is a highly significant limitation in the conclusions we can obtain from our results. Nevertheless, the effect observed after RuBiDopa uncaging is robust and we propose these findings as the preliminary stage from which further investigation can be founded. Future directions point to the replication of these results and additional experiments aiming to strengthen our conclusions and broaden our understanding about the role of DA in the modulation of cognitive processes in the prefrontal cortex.

## Author contributions

The study was designed by VA, RE, and SR. The chemical syntheses were performed by VA and RE. The experiments were performed by VA and AS. The analyses were performed by VA and RS. The figures were prepared by VA, RS, and SR. The manuscript was written by VA, RS, AT, and SR.

### Conflict of interest statement

The authors declare that the research was conducted in the absence of any commercial or financial relationships that could be construed as a potential conflict of interest.

## Supplementary material

Supplementary Figure 1 **Theta-phase modulates gamma oscillations in the medial Prefrontal Cortex**. Group data showing 3 experiments in Rat 1 **(upper panels)**, Rat 2 **(middle panels)** and Rat 3 **(lower panels)**. Spectrograms show that theta power is high in active waking and REM sleep periods, and it is low during quite wake. During active waking periods, there is a strong theta-gamma comodulation in medial prefrontal cortex. Spectrogram colorscale in mV^2^/Hz.

Supplementary Figure 2 **Statistical evaluation of comodulograms**. A surrogate distribution (see Methods section) of 300 comodulograms was constructed for both comodulograms shown in Figure 2B (panel showing theta—gamma coupling) and Figure 3C (100–150 s panel showing delta-gamma coupling). For each x-y entry, values lower than 0.975 of the surrogate distribution were set to zero (dark blue color in the heat-map); higher values were kept intact.

Supplementary Figure 3 **Comodulation during SWS**. Representative comodulogram calculated for a SWS epoch **(left panel)**. Delta-gamma comodulation is present, however notice a low effect and unstructured comodulation island. The modulation index for delta-gamma of this same epoch was analyzed while controlling for normalized delta amplitude **(right panel)**.

Supplementary Figure 4 **Delta and gamma power before and after laser onset. (A)** Power Spectrum Densities for delta (left) and gamma (right) bands. Solid line represents the mean of all the channels analyzed (*N* = 37) and dashed lines represent ±std. Note that delta (1–4 Hz) and gamma (60–110 Hz) power peaks before (black trace) and after (red trace) laser onset are the same. **(B)** Extraction of delta (left) and gamma (right) power for each animal. Each color represents one animal, error bars represent SEM (*N* = number of channels for each animal; orange: 14 channels; green: 13 channels; blue: 10 channels) and asterisks represent significant differences (*t*-test, *p* < 0.05).

Supplementary Figure 5 **Delta-gamma and theta-gamma comodulation for behavioral states**. Mean delta—gamma **(left panel)** and theta—gamma **(right panel)** comodulations were obtained for epochs around laser onset during either a delta- (quiet waking) or a theta-associated state (active waking), respectively. Same animals used in figure 4 and 5 (*N* = 3; different colors represent different animals).
