# Supplementary figures and images for: Dopamine Modulates Delta-Gamma Phase-Amplitude Coupling in the Prefrontal Cortex of Behaving Rats
Source: Front Neural Circuits. 2017 May 9;11:29. doi: 10.3389/fncir.2017.00029 (PMC5422429; doi:10.3389/fncir.2017.00029)

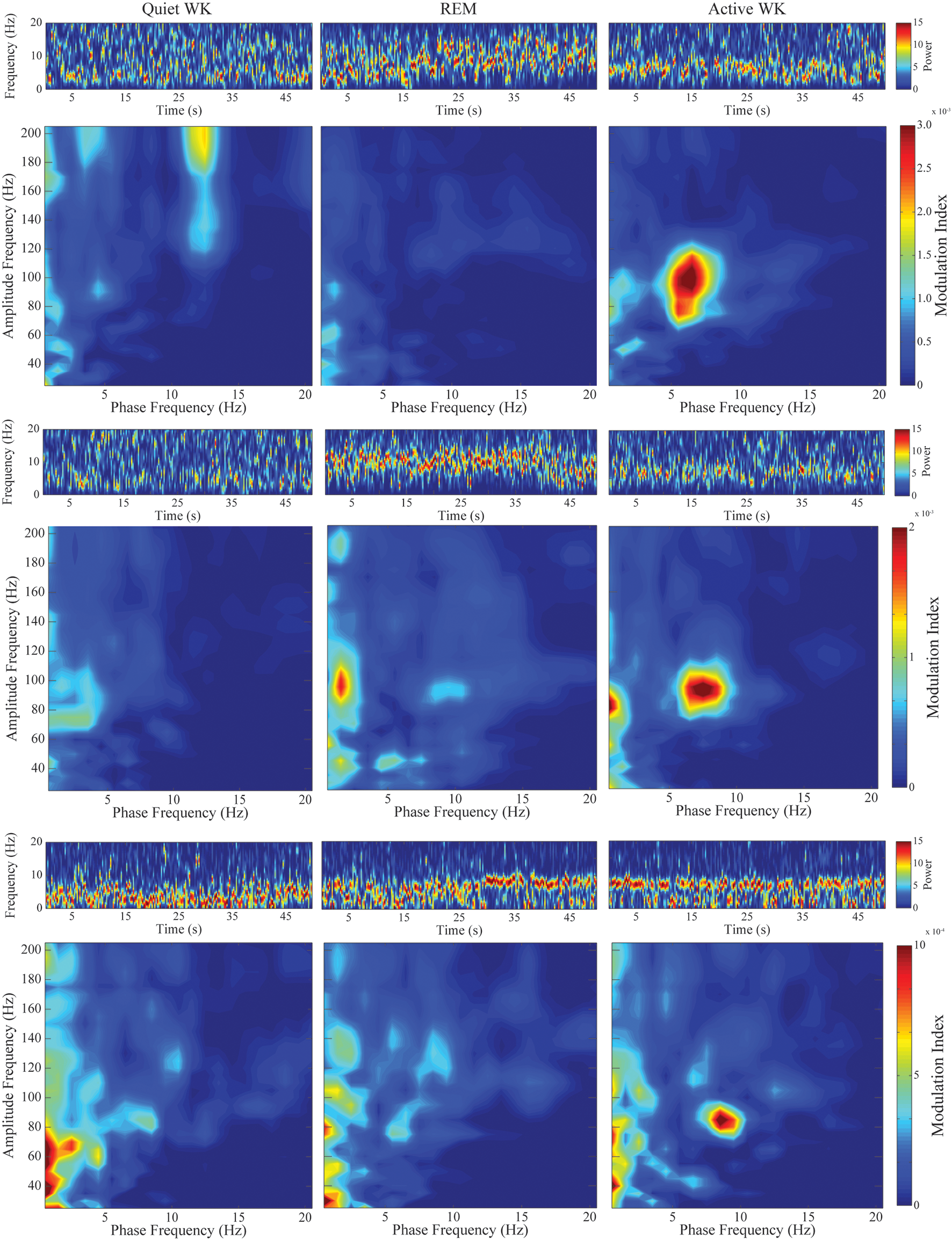

Supplement: Supplementary Figure 1 — Theta-phase modulates gamma oscillations in the medial Prefrontal Cortex. Group data showing 3 experiments in Rat 1 (upper panels), Rat 2 (middle panels) and Rat 3 (lower panels). Spectrograms show that theta power is high in active waking and REM sleep periods, and it is low during quite wake. During active waking periods, there is a strong theta-gamma comodulation in medial prefrontal cortex. Spectrogram colorscale in mV2/Hz. [file Image1.TIF]

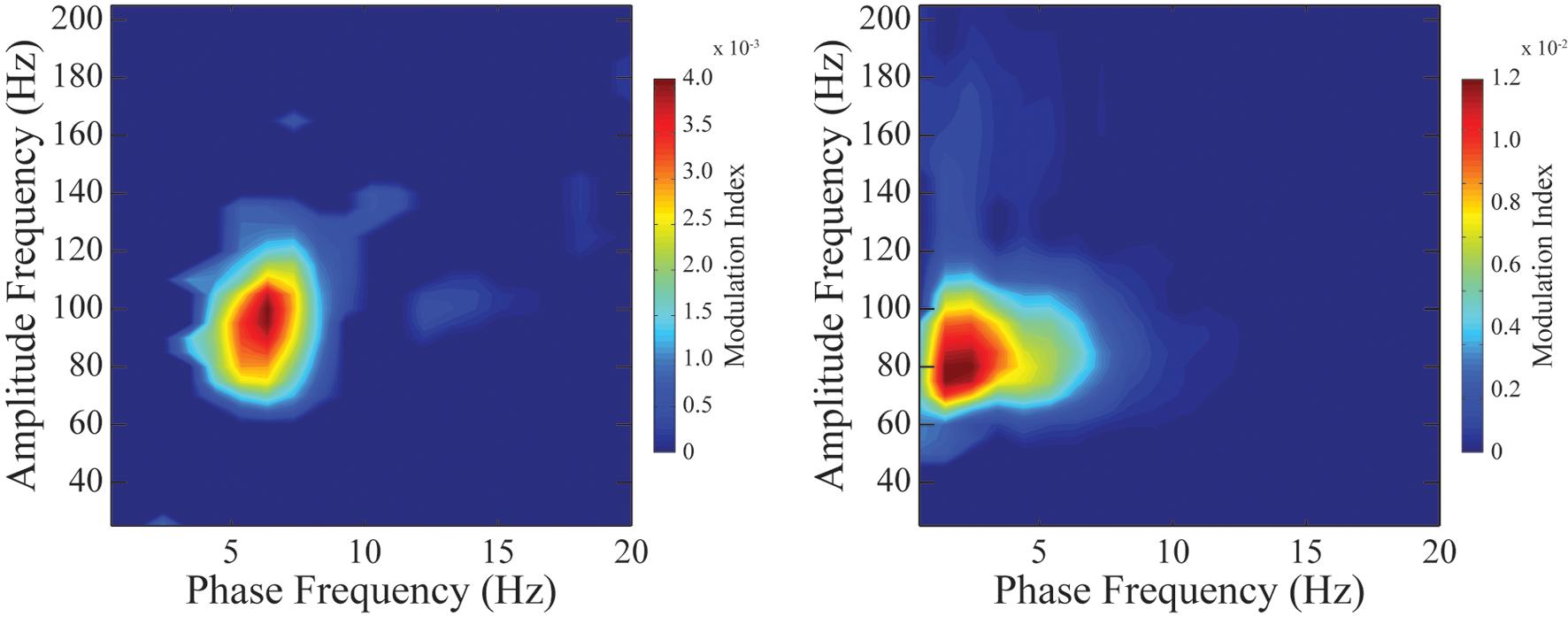

Supplement: Supplementary Figure 2 — Statistical evaluation of comodulograms. A surrogate distribution (see Methods section) of 300 comodulograms was constructed for both comodulograms shown in Figure 2B (panel showing theta—gamma coupling) and Figure 3C (100–150 s panel showing delta-gamma coupling). For each x-y entry, values lower than 0.975 of the surrogate distribution were set to zero (dark blue color in the heat-map); higher values were kept intact. [file Image2.TIF]

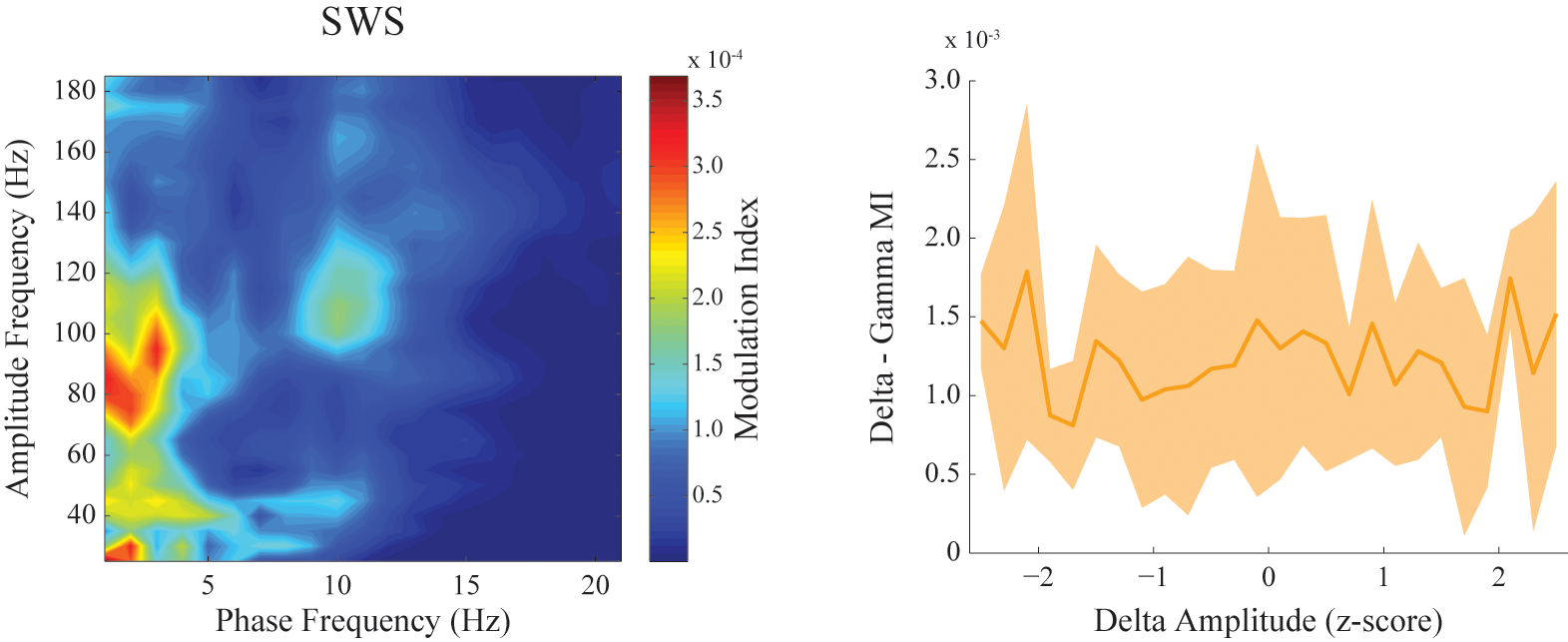

Supplement: Supplementary Figure 3 — Comodulation during SWS. Representative comodulogram calculated for a SWS epoch (left panel). Delta-gamma comodulation is present, however notice a low effect and unstructured comodulation island. The modulation index for delta-gamma of this same epoch was analyzed while controlling for normalized delta amplitude (right panel). [file Image3.TIF]

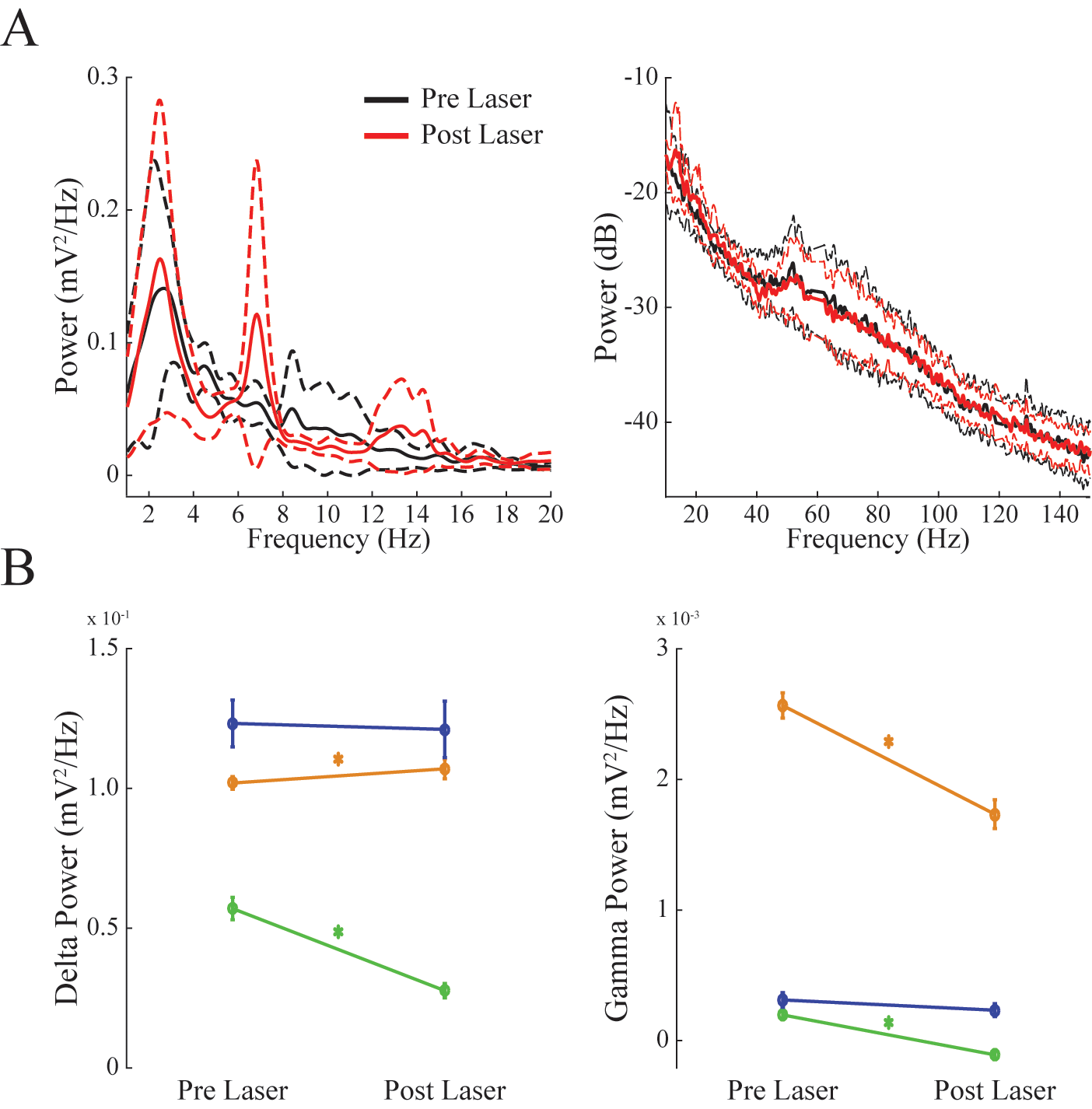

Supplement: Supplementary Figure 4 — Delta and gamma power before and after laser onset. (A) Power Spectrum Densities for delta (left) and gamma (right) bands. Solid line represents the mean of all the channels analyzed (N = 37) and dashed lines represent ±std. Note that delta (1–4 Hz) and gamma (60–110 Hz) power peaks before (black trace) and after (red trace) laser onset are the same. (B) Extraction of delta (left) and gamma (right) power for each animal. Each color represents one animal, error bars represent SEM (N = number of channels for each animal; orange: 14 channels; green: 13 channels; blue: 10 channels) and asterisks represent significant differences (t-test, p < 0.05). [file Image4.TIF]

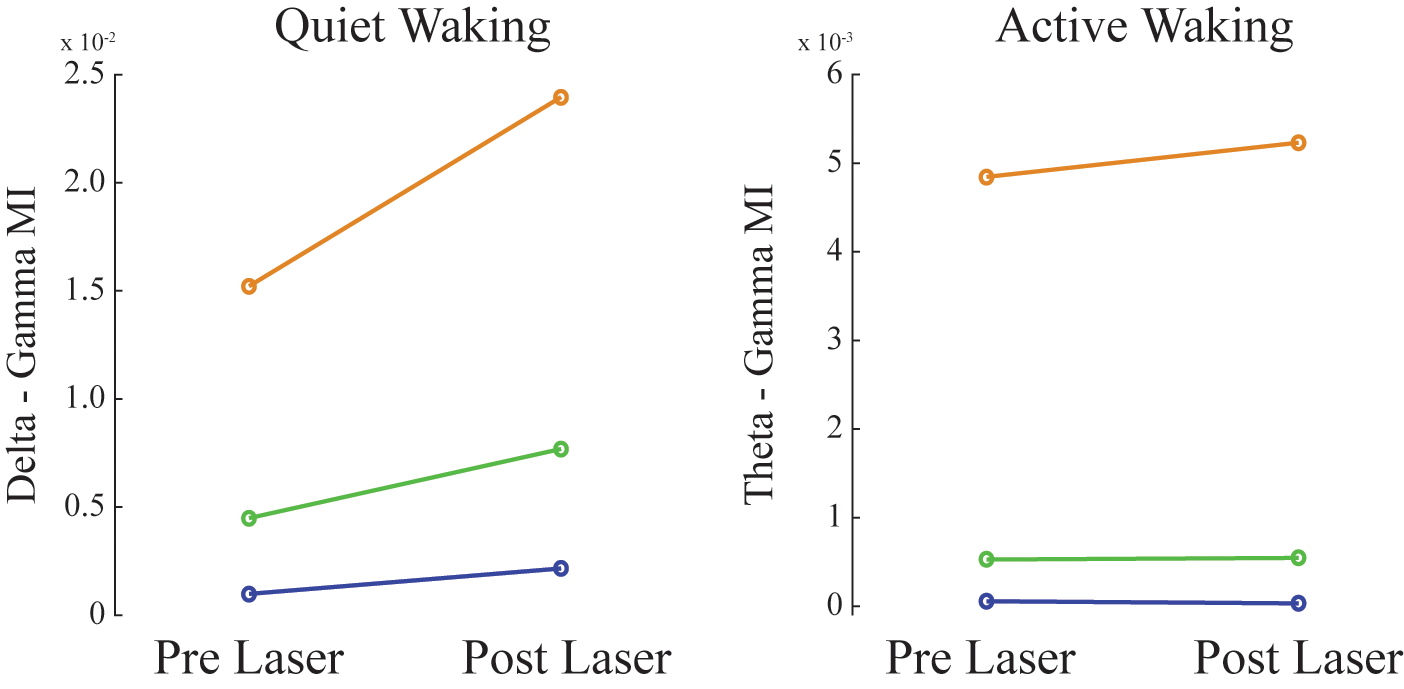

Supplement: Supplementary Figure 5 — Delta-gamma and theta-gamma comodulation for behavioral states. Mean delta—gamma (left panel) and theta—gamma (right panel) comodulations were obtained for epochs around laser onset during either a delta- (quiet waking) or a theta-associated state (active waking), respectively. Same animals used in figure 4 and 5 (N = 3; different colors represent different animals). [file Image5.TIF]
